# Physical activity, health-related fitness, and physical performance in children with acquired hypothalamic dysfunction

**DOI:** 10.1007/s00520-025-09361-5

**Published:** 2025-03-18

**Authors:** I. M. A. A. Van Roessel, J. Van Schaik, L. B. Kleinlugtenbelt, S. N. van Duijn, M. Burghard, T. Takken, W. J. E. Tissing, W. P. Bekkering, H. M. van Santen

**Affiliations:** 1 Department of Pediatric Endocrinology, Wilhelmina Children’s Hospital, University Medical Center Utrecht, Lundlaan 6, 3584 EA Utrecht, The Netherlands; 2 Division of Pediatric Neuro-Oncology, Department of Supportive Care, Princess Máxima Center for Pediatric Oncology, Heidelberglaan 25, 3584 CS Utrecht, The Netherlands; 3 Department of Exercise Physiology, Child Development & Exercise Center, Wilhelmina Children’s Hospital, University Medical Center Utrecht, Lundlaan 6, 3584 EA Utrecht, The Netherlands; 4 Department of Exercise Physiology, Exercise Center, Princess Máxima Center for Pediatric Oncology, University Medical Center Utrecht, Heidelberglaan 25, 3584 CS Utrecht, The Netherlands; 5 Division of Pediatric Oncology, University of Groningen, University Medical Centre Groningen, Hanzeplein 1, 9713 GZ Groningen, The Netherlands

**Keywords:** Childhood brain tumor survivors, Hypothalamic-pituitary dysfunction, Cardiorespiratory fitness, Physiotherapy, Lifestyle

## Abstract

**Purpose:**

Survivors of a pediatric suprasellar tumor may suffer from hypothalamic-pituitary dysfunction (HD), which may result in hypothalamic obesity (HO). The first step in HO treatment is lifestyle intervention (e.g. exercise). Our aim was to assess physical activity (PA), health-related fitness (HRF) and physical performance (PP) in a cohort of children with a suprasellar tumor.

**Methods:**

Retrospective study on a national cohort including all children with a suprasellar tumor who were referred to the physiotherapy department 2018—2022. Data was collected on: PA defined as minutes of Moderate-to-Vigorous Physical Activity (MVPA) and number of steps per day, HRF defined as body composition, VO_2_peak percentage of predicted, mean power, and muscle strength, and PP based on the 10-m walk and run test, time up and down the stairs, and time to rise from the floor.

**Results:**

Seventy-three children (mean age 11.09, mean body mass index SDS 2.36) were evaluated. In total, 24.1% reached the guideline of ≥ 60 min MVPA per day. The VO_2_peak percentage of predicted was 71.0% [IQR 57.0 – 82.8] and in 58.3% mean power was ≤ -2 SDS. Muscle strength was not decreased (median of -0.5 SDS). PP was found to be better than the norm.

**Conclusion and key findings:**

PA and HRF are decreased in children with HD, however PP was not decreased. This implies that no PP restrictions are present to engage in PA and that a lifestyle coach can be involved to improve PA and HRF in these children.

## Introduction

Suprasellar brain tumors account for 10% of all brain tumors amongst children [1]. Most commonly, suprasellar tumors are low grade, such as low grade glioma (LGG) or craniopharyngioma (CP) [2]. Treatment varies from chemotherapy (LGG) to surgery, sometimes with adjuvant radiotherapy [2, 3]. Survival rates for suprasellar tumors are high, but survivors may suffer from severe consequences of the tumor or its treatment because of damage to important structures located in this area [4]. One of the most severe and life disrupting sequelae is hypothalamic dysfunction (HD). HD can result in pituitary deficiencies, but also in reduced energy expenditure, temperature dysregulation, circadian rhythm disturbance, leptin resistance, disturbed satiety, hyperinsulinemia, and loss of initiative, all resulting in the development of hypothalamic obesity (HO) [5]. In addition, severe psychosocial disorders may be present as a consequence of damage to the connective circuits of the hypothalamus to the frontal lobe and the limbic system [6, 7]. HO results in increased cardio-vascular morbidity, higher mortality rates, and decreased quality of life [8].

HD is a multisystem morbidity and may be considered a chronic disease. Although some positive interventions have been reported [7, 9], these are only effective in a select group of patients and there is overall still no effective treatment for HO. The cornerstone for treatment of any obesity regardless of its entity should be optimization of lifestyle [10]. Within lifestyle, next to a healthy diet and adequate sleep, exercise is of great importance. Multiple factors in children treated for a suprasellar tumor may affect optimal physical activity (PA). Physical factors that can be influenced by interventions of the physiotherapist, such as health-related fitness (HRF) (includes body composition, cardiorespiratory fitness, mean power, and muscle strength) and physical performance (PP) (includes motor function) could contribute to the reduced activity levels. Also disease-related factors such as under-substitution of pituitary hormones (fear for hypocortisolism), vision loss, Body Mass Index (BMI), and psychosocial factors (e.g. initiative to be physically active) play a role [4, 11]. Only a few studies have reported on PA, HRF, or PP in patients with suprasellar tumors, reporting low levels of PA [11–13]. VO_2_ max (a reflection of cardiovascular function) in children with craniopharyngioma was reported to be 20% lower compared to healthy controls [14]. Conklin et al. found that motor abilities were significant worse in children with a suprasellar tumor [15].

Literature on PA, HRF, or PP often featured small sample sizes. We aimed, with a study conducted at a national pediatric oncology center including a relatively large population of suprasellar tumors, to get more insight into possible barriers contributing to low physical activity in children with suprasellar brain tumors. With improved insight, adequate advice and lifestyle interventions can be developed. For our aim, we assessed PA, HRF, and PP and the association with BMI in children with suprasellar tumors.

## Methods

### Patient characteristics

A retrospective chart evaluation was performed of all patients at risk for hypothalamic-pituitary dysfunction who were referred to the physiotherapist and clinical exercise physiologist in a national specialized center for pediatric oncology. Measurements of PA, HRF, and PP between 2018 and 2022 were included.

Inclusion criteria were age between 5 and 18 years and diagnosis of a suprasellar tumor. Patients were excluded when PA, HRF, or PP measurements were done under treatment of psychostimulants for treatment of HO, such as dextroamphetamine or methylphenidate.

### Data collection

#### Patient characteristics

Demographic, tumor- and treatment related characteristics, and anthropometric data (BMI Standard Deviation Score (SDS)) were extracted from medical records. Presence of overweight or obesity was determined by using the BMI cut-off points per age defined by Cole et al. [16] . Visual acuity disorders were categorized according to the definitions of visual impairment and blindness based on the International Statistical Classification of Diseases and Related Health Problems, Tenth Revision [17]: mild or no visual impairment (best corrected visual acuity (BCVA) ≤ 0.5 logMAR [Snellen fraction (SF) ≥ 20/70]), moderate visual impairment (BCVA > 0.5 to 1.0 logMAR [SF < 20/70 to ≥ 20/200]), severe visual impairment (BCVA > 1.0 to 1.3 logMAR [SF < 20/200 to ≥ 20/400]), and blindness (BCVA > 1.3 logMAR [SF < 20/400]). Visual field disturbances were scored as present if abnormalities (e.g. absolute or relative defects) were reported by the ophthalmologist [18].

#### Hypothalamic-pituitary disorders

Hypothalamic dysfunction and the hypothalamic syndrome were scored using the new diagnostic criteria for hypothalamic dysfunction by van Santen et al. [5]. The hypothalamic syndrome is considered the most severe form of hypothalamic dysfunction, characterized by impairment across multiple domains.

Presence of pituitary dysfunction was assessed. Any pituitary disorder was defined as any anterior pituitary disorder (including growth hormone deficiency (GHD), thyroid-stimulating hormone deficiency (TSHD), adrenocorticotropic hormone deficiency (ACTHD), luteinizing hormone deficiency (LHD)/follicle-stimulating hormone deficiency (FSHD), arginine vasopressin deficiency (AVP-D), or central precocious puberty (CPP)). Panhypopituitarism was scored if there was deficiency of all anterior hormones with or without DI.

#### Physical activity (PA)

PA was examined by the Actigraph GT3X accelerometer in minutes of Moderate-to-Vigorous Physical Activity (MVPA) per day and number of steps per day. The threshold to define MVPA was based on reference values according to age [19, 20]. Children were asked to wear the Actigraph GT3X accelerometer with a band around the hip or wrist for a week. The Actigraph GT3X is the adherent of the GT1M and is a valid and reliable tool for measuring PA [21]. The average of minutes MVPA per day and the average steps per day were calculated per participant. Results were compared to (inter)national guidelines [22, 23].

#### Health-related fitness (HRF)

The Bouchard model for the definition of HRF as a combination of body composition, muscular strength, cardiorespiratory fitness, and flexibility was used [24]. The following HRF components were collected in our study: body composition, cardiorespiratory fitness, mean power, and muscle strength [23].

Body composition was measured by height (wall-mounted measuring stick), weight (Tanita MC780, Tanita Corporation, Japan), and a Body Impedance Analysis (BIA) using the Bodystat 4000 (Bodystat Ltd, UK) or Tanita [25]. Fat-mass (%) and Fat-free mass (%) were collected. The BIA is a valid method for measuring body composition in obese children [26]. Percentiles for fat-mass were calculated according to sex-specific centile curves of McCarthy et al. [27].

Cardiorespiratory fitness was measured by the Peak Oxygen Uptake (VO_2_peak) using the Cardio Pulmonary Exercise Test (CPET). The measurement of VO_2_peak during a progressive CPET up to maximal exertion is widely considered the gold standard for assessing cardiorespiratory fitness [28]. The CPET was performed on a electronically braked cycle ergometer (Lode Corival, Lode BV, Groningen, the Netherlands). For the CPET, a ramp protocol was used, which is a valid and reliable procedure to measure VO_2_peak in children [29]. The VO_2_peak was presented as percentage of predicted for age and biological sex.

To measure Mean Power, the Muscle Power Sprint Test (MPST) was used [30, 31]. Mean Power was defined in watts and a SDS was computed for children older than 12 years, using norm values [30]. For children under 12 years of age, unstandardized data was presented, as no norm values are published to date.

Muscle strength was based on grip strength, using the Lode Held Dynamometer with the three-point grip protocol [32]. Grip strength was defined in kilograms and a SDS was computed using norm values [33].

#### Physical performance (PP)

PP was defined by exercise time on the 10-m walk test (10MWT), 10-m run test (10MRT), time up and down the stairs (TUDS), and time to rise from the floor (TRF) [34]. The 10MWT, 10MRT, TUDS, and TRF are functional mobility outcomes for measuring functional motor skills [34–36]. Performance times were defined in seconds and SDS were computed for children under 12 years using norm values [34]. Lower (or negative) SDS indicates a better performance at the test (as the child needed less seconds to perform). For children older than 12 years, unstandardized data was presented as no norm values are published to date.

### Statistical analysis

Data are presented as mean ± SD or median [range] for continuous data, depending on the distribution. Data are presented as percentages for categorical variables. Between-group differences were evaluated by Student’s T test for continuous data with a normal distribution, Mann–Whitney U test for continuous data with a skewed distribution, and by χ2 test or Fisher’s exact test for categorical data. To assess violation of normality distribution, QQ plot of the residuals and the Shapiro–Wilk’s test were employed. Additional to the descriptive statistics, a graphical display of the PA and HRF was developed.

To study possible risk factors on the outcome, univariate and multivariable linear regression analyses were performed. Independent variables to be included in the multivariable linear regression were selected by estimating first the univariate model and by considering the clinical relevance of each variable. A two-sided p-value of < 0.05 was considered statistically significant. Data were analyzed using SPSS version 27.

## Results

In total, 73 children were included in the study. Of the children, 50.7% were female (Table 1). CP was the most common suprasellar tumor with 46.6% (*n* = 34), other diagnoses were LGG (43.8%), and germinoma (8.2%). Mean age at tumor diagnosis was 6.34 years ± 3.97, and mean age at follow-up was 11.09 years ± 3.38. Of the 73 children, all exhibited at least one symptom of hypothalamic dysfunction and 61.6% were diagnosed with hypothalamic syndrome. For 9.6% of the children, the available data did not cover all domains required to determine the presence of hypothalamic syndrome. Of the 73 patients, 76.7% (*n* = 56) experienced any pituitary disorder and 52.1% had panhypopituitarism. In 15/71 (20.5%) moderate or severe loss of visual acuity (BCVA > 0.5 logMAR) or blindness of both eyes was present. In 50/67 (74.6%) of the patients, a visual field disturbance (either absolute of relative defects) was present at moment of physical evaluation. Mean BMI SDS was 2.36 ± 1.17.

**Table 1:** Patient characteristics

Total group (*n* = 73, 100%)	
Female, *n* (%)	37 (50.7)
Mean age at tumor diagnosis, years (SD)	6.34 ± 3.97
Mean age at follow-up, years (SD)	11.09 ± 3.38
Median follow-up time, years (IQR)	3.43 (2.00—8.54)
Type of suprasellar tumor	
Craniopharyngioma	34 (46.6)
Low grade glioma	32 (43.8)
Germinoma	6 (8.2)
Unknown tumor*	1 (1.4)
Mean height SDS at evaluation	−0.73 ± 1.47
Mean weight SDS at evaluation	1.73 ± 1.57
Mean BMI SDS at evaluation	2.36 ± 1.17
Visual capacities at FU (*n* = 71)*	
- Normal or mild visual acuity	56 (78.9)
- Decreased visual acuity (moderate/severe)	11 (15.5)
- Blindness	4 (5.6)
- Visual field impairments *(n* = *67)*	50 (74.6)
Hypothalamic-pituitary deficiencies at diagnosis or follow-up	
Hypothalamic dysfunction	73 (100.0)
Hypothalamic syndrome	45 (61.6)
Anterior pituitary deficiencies	56 (76.7)
Posterior pituitary deficiencies (AVP-D)	39 (53.4)
CPP	15 (20.5)
Panhypopituitarism with AVP-D	38 (52.1)

*BMI* Body mass index; *SDS* Standard deviation score; *FM* Fat mass; *FU* Follow-up; *DI* Diabetes insipidus; *CPP* Central precocious puberty. Numbers are presented as *n* (%) or mean ± SDS^*^Normal or mild visual impairment (BCVA ≤ 0.5 logMAR [SF ≥ 20/70]), moderate visual impairment (BCVA > 0.5 to 1.0 logMAR [SF < 20/70 to ≥ 20/200]), severe visual impairment (BCVA > 1.0 to 1.3 logMAR [SF < 20/200 to ≥ 20/400]), and blindness (BCVA > 1.3 logMAR [SF < 20/400]) [17]. Visual field examination was performed using age-adapted testing and was scored as impaired if abnormalities (e.g. absolute or relative visual field defects) were reported by the ophthalmologist [18]. ^*^ In one of the patients it was not possible to classify the tumor based on biopsy

### Physical activity, health-related fitness, and physical performance

**Fig. 1:**
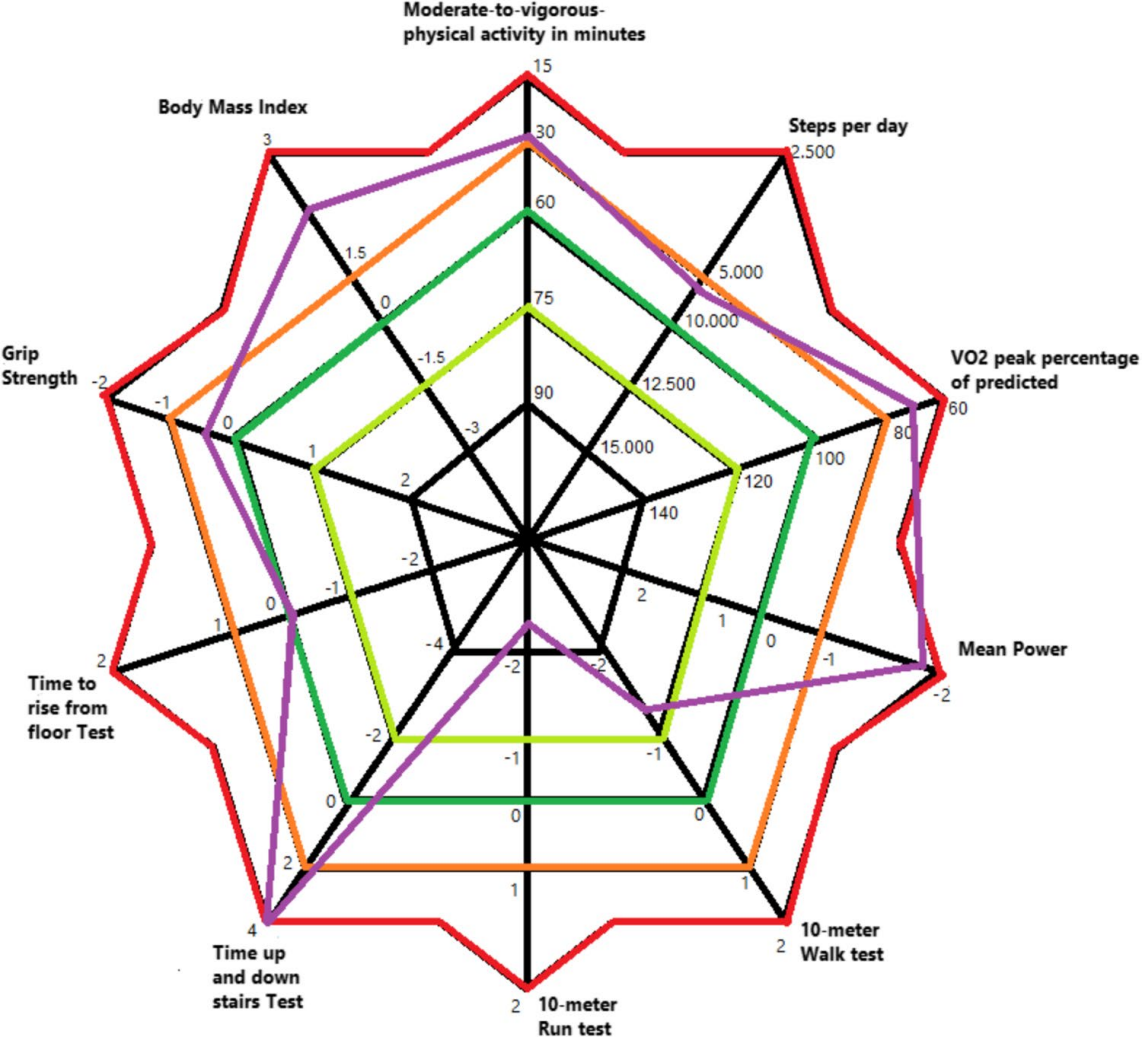
Graphical display of physical activity, health related fitness, and physical performance. Purple line represents the mean or median result of each test in the total cohort (n = 73). The outer lines (red) represent the poorest scores compared to normative values. For example, the outer line of BMI represents severe obesity (3 SDS) whereas the inner line (green and black) of BMI represent normal weight and underweight, respectively. The outer line of the grip strength, time to rise from floor test, time up and down stairs test, 10 m run test, 10 m walk test indicate a lesser test performance

The combined data on PA, HRF, and PP are depicted in Fig. 1 and Table 2.

**Table 2:** Results of physical activity, health related fitness, and physical performance

	*N* (%)	Median	Range		Median SDS or %	Range	
			Min	Max		Min	Max
Physical activity							
MVPA (minutes)	54 (74.0)	34.4	0.0	125.3	13 (24.1) ≥ 60 min		
Steps per day (per 1000)	60 (82.2)	5.9	0.3	17.2	10 (16.7) ≥ 10.000		
Health related fitness							
BMI SDS	73 (100)	23.6	15.2	40.5	2.36	−0.68	4.91
Weight classification							
Normal weight	16 (21.9)						
Overweight	32 (43.8)						
Obesity	25 (34.2)						
FM (%)	70 (95.9)	15.4	2.9	54.4	32.9	9.4	48.5
VO_2_peak (% of pred)	60 (82.2)				71.0	37.0	107.0
Mean power (watts)							
Age < 12 years	33 (80.5)	77.5	15.6	262.8	*	*	*
Age ≥ 12 years (28)	24 (75.0)	181.7	42.1	399.2	−1.9	−3.4	−0.5
Grip strength (kg)							
Dominant	62 (84.9)	19.0	4.8	42.3	−0.5	−2.3	4.0
Non-dominant	61 (83.6)	16.7	3.7	45.6	−0.6	−2.9	3.7
Physical performance							
TRF (sec)							
Age ≤ 12 years (32)	37 (80.4)	1.3	0.5	5.7	0.1	−1.1	10.3
Age > 12 years	17 (63.0)	1.9	1.0	5.3	*	*	*
10MWT (sec)							
Age ≤ 12 years (32)	43 (93.5)	7.5	3.9	30.5	−1.3	−3.3	10.7
Age > 12 years	21 (77.8)	7.1	5.2	8.7	*	*	*
10MRT (sec)							
Age ≤ 12 years (32)	42 (91.3)	3.2	2.2	21.3	−2.2	−3.8	24.7
Age > 12 years	23 (85.2)	3.2	2.6	12.3	*	*	*
TUDS (sec)							
Age < 6 years	3 (100.0)	16.3	11.8	17.9	*	*	*
Age ≥ 6—15 years (32)	48 (85.7)	8.4	4.8	58.0	4.0	−2.4	79.6
Age ≥ 15 years	10 (13.7)	6.7	6.0	38.0	*	*	*

^*^ No norm values available *MVPA* Moderate-to-vigorous physical activity; *FM* Fat mass; *VO*_*2*_ Peak oxygen uptake; *TRF* Time to rise from the floor; *10MQT* 10-m walk test; *10MRT* 10-m run test; *TUDS* Time up and down the stairs

### Physical activity (PA)

Of the 73 children, in 54 (74.0%) MVPA was measured. Multiple guidelines (including WHO) recommend a minimum of 60 min of MVPA every day for school-age children [22]. The median MVPA was 34.43 min per day [IQR 14.68 – 59.89]. Of children aged 4–12 years 37.1% reached the national guideline for MVPA and 0.0% in children aged 12–16 years. In the Netherlands, 55.4%−62.3% of the healthy children between 4–12 years meet the 60 min MVPA requirement and 34.8%−44.1% of the healthy children between 12–16 years [37].

The number of steps has been recommended to be an average of 12.000–16.000 per day for boys and 10.000–13.000 for girls [23]. In 60 children (82.2%) that were measured for step count, the median amount of steps per day was 5892 [IQR 4404 – 8412]. Of these 60, ten children (16.7%) had an average of 10000 steps or higher per day and 25 children (41.7%) had an average step count between 5000–10000 steps per day.

### Health-related fitness (HRF)

#### Body composition

Of patients who were measured for body composition (*N* = 70), mean height SDS and weight SDS at time of measurement were −0.73 ± 1.47 and 1.73 ± 1.57, respectively. Mean BMI SDS was 2.36 ± 1.17. In total, 21.9% (*n* = 16) scored as normal weight, 43.8% (*N* = 32) scored as overweight, and 34.2% (*N* = 25) scored as obese. The mean percentage of Fat-Mass was 32.87% ± 8.30. Of these, 55 (78.6%) scored above the 90th percentile for fat mass adjusted for age and sex.

#### Cardiorespiratory fitness

CPET was performed in 60 patients. Median percentage of predicted VO_2_peak was 71.0% [IQR 57.0 – 82.8].

#### Mean muscle power

In total 57 patients performed the MPST. Of 24 children aged 12 years or older, mean muscle power SDS was −1.92 [IQR −2.60 – −1.23]. In 14/24 (58.3%) mean muscle power SDS was ≤ −2 SDS.

#### Muscle strength

The median SDS for grip strength of the dominant hand (*n* = 62) was −0.51 [IQR −1.14 – 0.29] and for the non-dominant hand (*n* = 61) −0.55 [IQR −1.37 – 0.12]. Of 16/62 (25.8%) children, the median SDS was ≤ −1 and in 3/62 (4.8%) ≤ −2. For the non-dominant hand, this was 26/61 (42.6%) and 5/61 (8.2%).

### Physical performance (PP)

Physical performance was tested using the TRF (*n* = 54), 10MWT (*n* = 64), 10MRT (*n* = 65), and TUDS (*n* = 65). Median SDS scores are included in Table 2. Only for children lower than 12 years of age norm values were available. For the TRF, 12/37 (32.4%) scored ≥ + 1 SDS and 5/37 (13.5%) ≥ + 2 SDS (reflecting worse performance). In total 16 children (43.2%) had a lower score than 0 SDS (reflecting better performance). For the 10MWT, three out of 43 patients (7.0%) scored ≥ + 1 SDS, and one patient (2.3%) scored ≥ + 2 SDS. In total 38 children (88.4%) had a lower score than 0 SDS, of whom 29 (67.4%) a lower score than −1 SDS and ten (23.2%) a lower score than −2 SDS. For the 10MRT, two patients (4.8%) scored ≥ + 2 SDS, and 37 out of 42 patients (88.1%) scored lower than 0 SDS, of whom 34 patients lower than −1 SDS (80.1%) and 25 lower than −2 SDS (59.5%). For TUDS, 39/48 (81.3%) scored above + 1 SDS and 32/48 (66.7%) scored above + 2 SDS. One patient scored lower than −2 SDS (2.1%).

### HRF and PP in relation to PA (Table 3)

**Table 3:** Results of univariate and multivariable analyses of PA in relation to HRF and PP

	Univariate				Multivariable (*N* = *30*)			
	B	95% CI		*P*-value	B	95% CI		*P*-value
Mean steps per week (per 1000 steps)								
VO_2_ peak percentage of predicted	0.055	0.006	0.104	0.028*	0.052	−0.013	0.117	0.112
Mean power SDS	0.698	−0.959	2.355	0.391				
Grip strength SDS	−0.093	−0.963	0.778	0.832				
TRF	−0.460	−0.969	0.049	0.075				
10MWT	−0.612	−1.698	0.473	0.259				
10MRT	−1.096	−1.737	−0.454	0.001*	−0.704	−2.124	0.717	0.318
TUDS	−0.101	−0.195	−0.007	0.036*	−0.037	−0.210	0.135	0.662

^*^ Indicates a significant *P*-value (< 0.05)

In the univariate linear regression, VO_2_peak percentage of predicted (B = 0.055; 95% CI 0.006 – 0.104, *p* = 0.028) was significantly associated to mean steps per week (per 1000 steps). Grip strength SDS and mean power SDS were not significantly associated. The 10MRT (B = −1.096; 95% CI −1.737 – −0.454, *p* = 0.001) and TUDS (B = −0.101; 95% CI −0.195 – −0.007, *p* = 0.036) were both univariately significantly associated to mean steps per week. The 10MWT and TRF were not significantly associated. In multivariable analyses, VO_2_peak percentage of predicted, 10MRT, and TUDS, were not significantly related with mean steps per week in Table 3.

### PA, HRF, and PP in relation to BMI SDS (Table 4)

**Table 4:** Results of univariate and multivariable analyses of BMI SDS in relation to PA, HRF, and physical performance

	Univariate				Multivariable (*N* = *30*)			
	B	95% CI		*P*-value	B	95% CI		*P*-value
BMI SDS								
Mean steps per week (per 1000 steps)	−0.088	−0.171	−0.004	0.040*	−0.065	−0.200	0.070	0.328
Mean MVPA per day	−0.002	−0.013	0.008	0.681				
VO_2_ peak percentage of predicted	−0.004	−0.021	0.014	0.685	0.012	−0.012	0.036	0.313
Mean power SDS	−0.309	−0.672	0.055	0.092				
Grip strength SDS	0.251	−0.018	0.519	0.067	0.072	−0.302	0.447	0.692
TRF	0.082	−0.141	0.304	0.461				
10MWT	−0.004	−0.206	0.198	0.967				
10MRT	0.000	−0.094	0.095	0.996				
TUDS	0.054	0.034	0.074	< 0.001*	0.076	0.019	0.134	0.011*
Moderate/severe impairment of visual acuity or blindness	0.451	−0.218	1.122	0.183	0.400	−0.756	1.556	0.480
Pituitary deficiency	−0.201	−0.861	0.459	0.545	0.350	−0.576	1.277	0.441
Hypocortisolism	−0.490	−1.061	0.081	0.092	−0.469	−1.383	0.445	0.299

^*^ Indicates a significant *P*-value (< 0.05)

In the univariate linear regression, mean steps per week and TUDS were significantly associated to BMI SDS. Mean MVPA per day, VO_2_peak percentage of predicted, mean power SDS, grip strength SDS, TRF, 10MWT, 10MRT, impaired visual acuity, or pituitary deficiency were not associated to BMI SDS.

In multivariable regression analysis, TUDS (B = 0.076; 95% CI 0.019 – 0.134,* p* = 0.011) was significantly associated to BMI SDS. Mean steps per week, VO_2_peak percentage of predicted, grip strength SDS, moderate/severely impairment of visual acuity or blindness, pituitary deficiency, and hypocortisolism were not significantly associated to BMI SDS in Table 4.

## Discussion

Our results show that children after treatment for a suprasellar tumor have decreased physical activity and health-related fitness, whereas they do not have muscular restrictions or lack the ability to perform exercise (normal physical performance). These findings are important and indicate that these children may benefit for guidance of a lifestyle coach rather than a physiotherapist. Hypothalamic obesity greatly influences quality of life [7]. The first step in HO treatment is optimization of lifestyle and with increased knowledge upon the barriers that prohibit such children to be active, better lifestyle programs can be developed.

A correlation between PA and components of HRF has been examined in healthy children. Nevill et al. found an association between VO_2_ peak and PA in healthy children [38]. A lack of PA has been widely recognized as an important factor in developing health problems and chronic diseases and especially contributes to worsening BMI in individuals with overweight [39]. Therefore, the low PA in children with a suprasellar tumor is a serious problem and may lead to an increased risk of future health problems and multimorbidity in later life. Indeed, we could show that cardiorespiratory fitness was decreased, with the median VO_2_peak percentage being 71.0% of predicted (reference value of the lower limit 80% of predicted [28]). Future physical exercise programs should therefore focus on improving cardiorespiratory fitness in these patients, such as introducing high intensity fitness programs for children. Before such programs can be developed, more insight is needed in its facilitators and barriers, for example whether low PP is a barrier for performing exercise.

It was remarkable that the results of the TRF, 10MWT, and 10MRT in our cohort were normal on average and for the 10MWT and 10MRT, results were even better compared to healthy peers. An explanation for the fact that the median TUDS SDS was reduced, might be a high percentage of visual field and acuity deficits in our cohort in combination with a high BMI (stair running is a weight bearing activity). The grip strength was not reduced, which corresponds with a previous finding that children with an increased weight are stronger because their body weight requires more muscle strength to move against gravity during the day [40].

In children with HD, loss of initiative may play a substantial role in low PA and low HRF. The hypothalamus plays an essential role in motivation, initiating an activity, and coming up with and achieving self-set goals [7]. The low PA, despite meeting the conditions to perform exercise, may largely be explained by this hypothalamic initiative loss, which is important to address to improve PA and HRF. In our experience, when encouraged, most children enjoy performing exercise.

This study has some limitations. In most cases, the reason for referral to the physiotherapy department was an increased BMI or the need for help with PA planning. This may have created a selection bias as less affected children with a suprasellar brain tumor were mostly not referred. However, although our evaluation may thus not represent all children with HD following suprasellar tumor treatment, this evaluation is of great value as the results gives us more insight into PA, HRF, and PP in affected children in this specific population. Unfortunately, not all scores could be standardized because of the lack of normative values. Therefore, we could not draw conclusions on all outcomes for specific age categories.

In conclusion, results of this study show that PA and HRF are decreased in children with a suprasellar tumor at risk for hypothalamic dysfunction, but PP, reflected as performance on motor function tests, is not impaired. This indicates that children with HO are not limited by lack of PP to exercise and to meet the recommendations for healthy PA. Of course, other barriers must be addressed such as increasing the hydrocortisone dose when performing high intensity training or sporting in a safe environment in case of visual impairments. One of the cornerstones in management of HO must be improvement of PA and HRF. Improved cardiorespiratory health and muscle mass will increase energy expenditure and thus decrease BMI. Given the intact physical performance, but often lack of initiative with psychological barriers, the ideal way to improve activity for these children may be via an lifestyle coach (combined lifestyle intervention) in the home environment.

## Data Availability

The data that support the findings of this study are not openly available due to reasons of sensitivity and are available from the corresponding author upon reasonable request.

## Abbreviations

ACTHD: Adrenocorticotropic hormone deficiency
AVP-D: Arginine vasopressin deficiency
BCVA: Best corrected visual acuity
BIA: Body impedance analysis
BMI: Body mass index
CP: Craniopharyngioma
CPET: Cardio pulmonary exercise test
CPP: Central precocious puberty
FM: Fat mass
FSH: Follicle-stimulating hormone deficiency
GHD: Growth hormone deficiency
HD: Hypothalamic dysfunction
HO: Hypothalamic obesity
HRF: Health-related fitness
LGG: Low-grade glioma
LH: Luteinizing hormone deficiency
MPST: Muscle power sprint test
PA: Physical activity
REE: Resting energy expenditure
SDS: Standard deviation score
TSHD: Thyroid-stimulating hormone deficiency
TRF: Time to rise from the floor
TUDS: Time up and down the stairs
VO_2_: Volume oxygen uptake
10MWT: 10-Meter walk test
10MRT: 10-Meter run test
MVPA: Moderate-to-vigorous physical activity
